# Interleukin-6 is a mediator of therapeutic efficacy in acute lung injury

**DOI:** 10.1093/ajrccm/aamag213

**Published:** 2026-04-28

**Authors:** Lina Kramer, Carolyn S Calfee, Daniel F McAuley, Cecilia O’Kane, Jurjan Aman, Erik Duijvelaar, Evangelos J Giamarellos-Bourboulis, Nikolaos Antonakos, Martijn W Heymans, Lieuwe D J Bos

**Affiliations:** Intensive Care, Amsterdam UMC, The Amsterdam Institute for Infection and Immunity, University of Amsterdam, Amsterdam, The Netherlands; Division of Pulmonary, Critical Care, Allergy and Sleep Medicine, Departments of Medicine and Anesthesia, University of California, San Francisco, San Francisco, CA, United States; Wellcome-Wolfson Institute for Experimental Medicine, School of Medicine, Dentistry and Biomedical Sciences, Queen’s University Belfast, Belfast, United Kingdom; Wellcome-Wolfson Institute for Experimental Medicine, School of Medicine, Dentistry and Biomedical Sciences, Queen’s University Belfast, Belfast, United Kingdom; Department of pulmonary medicine, Amsterdam UMC, Amsterdam Cardiovascular Sciences, VU University, Amsterdam, The Netherlands; Department of pulmonary medicine, Amsterdam UMC, Amsterdam Cardiovascular Sciences, VU University, Amsterdam, The Netherlands; 4th Department of Internal Medicine, National and Kapodistrian University of Athens, Medical School, Athens, Greece; 4th Department of Internal Medicine, National and Kapodistrian University of Athens, Medical School, Athens, Greece; Department of Epidemiology and Biostatistics, Amsterdam UMC, Amsterdam Public Health Research Institute, VU University, Amsterdam, The Netherlands; Intensive Care, Amsterdam UMC, The Amsterdam Institute for Infection and Immunity, University of Amsterdam, Amsterdam, The Netherlands

**Keywords:** inflammatory response, biological mechanisms, surrogate outcomes, longitudinal mediation analysis

## Abstract

**Rationale:**

Inflammation in acute respiratory distress syndrome (ARDS) and COVID-19 causes disruption of the alveolar-capillary barrier and tissue damage, which has been associated with increased mortality. Elevated pro-inflammatory plasma biomarker (specifically IL-6) levels reflect the degree of systemic inflammation and are linked to severity of lung injury. Understanding the causal pathway between inflammation, interventions, and patient outcomes could improve clinical care.

**Objectives:**

To assess IL-6 as a mediator of effective interventions in ARDS and COVID-19.

**Methods:**

We leveraged individual patient data from 5 large randomized controlled trials. Interventions were imatinib, anakinra, low tidal volume ventilation, high positive end–expiratory pressure (PEEP), and simvastatin. We evaluated IL-6 as a mediator for the effectiveness of interventions on 28-day survival with joint modeling. Additionally, we analyzed the role of other pro-inflammatory markers (IL-8, tumor necrosis factor receptor 1, and C-reactive protein). The association between IL-6 and mortality was meta-analyzed using a random effects model.

**Measurements and Main Results:**

For 2563 patients, IL-6 measures were available at baseline and at least 1 other timepoint. Interleukin-6 mediated the effects of imatinib, anakinra, and low tidal volume on mortality. Higher PEEP and simvastatin did not alter IL-6 trajectories. In all studies, there were associations between higher IL-6 concentrations and increased mortality over 28 days (pooled hazard ratio [HR] for a log10-unit increase = 4.83, 95% CI, 3.50-6.66).

**Conclusions:**

The mortality benefit by anakinra and imatinib in COVID-19 and low tidal volume ventilation in acute lung injury may operate through resolution of inflammation defined by a reduction in plasma IL-6 levels.

At a Glance Commentary **Scientific Knowledge on the Subject:** Despite decades of trials in acute respiratory distress syndrome (ARDS), few interventions have demonstrated consistent mortality benefit, and the molecular pathways linking effective treatments to recovery remain poorly understood. Elevated plasma interleukin-6 (IL-6) is associated with ARDS severity and mortality, and IL-6 pathway modulation improves outcomes in COVID-19. However, whether resolution of inflammation, reflected by IL-6 dynamics, lies on the causal pathway between effective interventions and survival across ARDS and COVID-19 therapies has not been systematically evaluated. Early-phase trials and clinical decisions rely on surrogates with limited prognostic evidence.

**What This Study Adds to the Field:** In this individual patient data analysis of five randomized trials (n=2,563; COUNTER-COVID, SAVE-MORE, ARMA, ALVEOLI, HARP-2), joint modeling of longitudinal biomarkers and survival was used to evaluate IL-6 as a treatment-effect mediator. The mortality benefits of imatinib, anakinra, and low tidal volume ventilation were each consistent with mediation through IL-6 reduction, whereas higher PEEP and simvastatin did not alter IL-6 trajectories. Higher IL-6 was consistently associated with increased 28-day mortality across cohorts. The findings support IL-6 as a candidate surrogate for monitoring treatment effectiveness in COVID-19 and ARDS, with potential utility for trial design and patient-level therapy evaluation.

## Introduction

Patients with acute respiratory distress syndrome (ARDS) develop diffuse, inflammatory, protein-rich pulmonary edema resulting in severe acute respiratory failure.^1^ Acute respiratory distress syndrome affects approximately 10% of patients admitted to the intensive care unit and carries a mortality rate of around 40%.^2^ Decades of preclinical experiments have provided evidence for numerous potentially beneficial interventions, but clinical trials in unselected ARDS populations have largely failed to demonstrate consistent benefit.^3^ More recently, advances in molecular phenotyping and interventions targeted at SARS-CoV-2–induced respiratory failure suggest that a mortality reduction can be achieved when a more homogeneous subgroup is targeted.^1^^,^^4-6^ Despite these advancements, the underlying molecular pathways toward recovery remain poorly understood.

Acute respiratory distress syndrome and COVID-19 are distinct illnesses with patients from heterogeneous populations, but they may share common pathways to recovery. Exuberant pro-inflammatory response is a key phenomenon in the pathophysiology of ARDS. Interleukin-6 is a pleiotropic cytokine that plays a central role in the transition between innate and adaptive immune responses. Excessive IL-6 production amplifies inflammatory response and consequently leads to tissue damage. In ARDS, the plasma concentration of IL-6 and its temporal dynamics are strongly associated with mortality, and IL-6 is an important predictor of allocation to the hyperinflammatory molecular phenotype.^7-9^ In COVID-19, modulation of the IL-6 pathway through inhibition of the receptor has shown to improve clinical outcomes.^8^ Previously, mediation analysis demonstrated that imatinib reduced mortality through lowering IL-6 plasma concentration.^7^

Trial design and clinical patient care could be significantly improved if the causal pathway between effective interventions and improved outcomes were better understood. Currently, early phase ARDS studies frequently rely on surrogate endpoints such as gas exchange, pulmonary edema, or extra-pulmonary organ dysfunction, despite the lack of evidence supporting the predictive value of these endpoints, or even contradictory results.^10-12^ In patient care, physicians need to rely on similar surrogates to evaluate treatment effects and encounter the same obstacles resulting in a lack of objective criteria for treatment failure and need for escalation. Resolution of inflammation could be a key intermediate disease state toward recovery and thereby function as a surrogate in early phase studies and evaluation of treatment efficiency in individual patients.

In this individual patient data analysis of randomized controlled trials (RCTs), we applied mediation analysis to elucidate the role of IL-6 as a surrogate marker for inflammation in the pathway toward recovery. We hypothesized that resolution of inflammation is a common pathway toward recovery in ARDS and COVID-19. Beyond IL-6, we evaluate the roles of plasma IL-8, C-reactive protein (CRP), and tumor necrosis factor receptor 1 (TNFR1). Our analysis leveraged data from 5 RCTs to better understand the dynamic interactions between different interventions and outcomes through resolution of inflammation.

Some of these results have been previously reported in the form of an abstract.^13^

## Methods

We follow the AGReMA (A Guideline for Reporting Mediation Analyses) statement for reporting mediation analyses of randomized trials.^14^

### Study design and inclusion criteria

This study is an analysis of individual patient clinical and biological data from the 5 RCTs: COUNTER-COVID, SAVE-MORE, ARMA, ALVEOLI, and HARP-2.^15-19^ The target population was patients with ARDS or COVID-19–related respiratory failure. Detailed descriptions of patient selection, study designs, information on ethical approval, and patients informed consent for each trial can be found in the corresponding original publications. Specifically, these 5 RCTs were included in this study because, for each of them, IL-6 measures from at least 2 timepoints were available. IL-8, TNFR1, and CRP were not measured in each study and could therefore only be analyzed in step with availability.

### Measurements

The primary outcome was survival time until day 28. Survival time to day 90 was investigated in a secondary analysis. The mediators were defined as patient-specific IL-6, IL-8, TNFR1, and CRP trajectories over the first week after random assignment. The interventions were treatment with imatinib (COUNTER-COVID), anakinra (SAVE-MORE), ventilation with low tidal volumes (ARMA), and high positive-end expiratory pressure (ALVEOLI), and treatment with simvastatin (HARP-2).

### Effects of interest

This mediation analysis investigates the underlying inflammatory mechanisms for the effectiveness of the 5 interventions on patient survival over 28 days. For each trial, total intervention effects were estimated and analytically separated into direct and indirect effects. Indirect effects refer to treatment effects that work through modulation of pro-inflammatory biomarker response. Direct effects refer to treatment effects that were through all other pathways (ie, not explained by biomarker response). To this end, we estimated the intervention effects on biomarker response over time, the associations between biomarkers and survival, and the intervention effect on survival when the biomarker was controlled for. The mediation analyses were conducted separately for each of the included trial populations. Of additional interest was the overall association between IL-6 concentration and mortality risk across studies. As a secondary analysis, we were interested in the same effects for the 90-day endpoint. Figure S2 includes an illustration of confounding assumptions.

### Statistical analysis

#### Stratification

In the available ARDS studies ALVEOLI and HARP-2, we had molecular phenotype allocation available through previous latent class analyses.^4^^,^^5^ For these studies, we stratified analyses for hypo- and hyperinflammatory phenotypes, as higher positive end–expiratory pressure (PEEP) and simvastatin have previously been shown to have differential treatment effects in these phenotypes.^4^^,^^5^

#### Mediation analysis

For each of the trial datasets and predefined subgroups, we evaluated the total effect of the intervention on 28-day mortality using Cox proportional hazards models. Mediation analysis was used to decompose the total treatment effects into direct and indirect effects.

To assess direct and indirect effects, we adopted the approach by van Oudenhoven and colleagues^20^ for mediation analysis using joint modeling of longitudinal and time-to-event data. A detailed illustration of the approach is provided in Supplement 2. In short, joint modeling allowed us to assess intervention-biomarker and biomarker-mortality relationships over time by combining 2 submodels for each trial: one submodel for the survival outcome and one submodel for the longitudinal biomarker (ie, the potential mediator). For each biomarker in each trial, we specified linear mixed models, including fixed effects for time and an interaction between time and the intervention, random intercepts for individual patients, and random slopes for time. We modeled patient survival outcomes with relative risk models including the multiplicative effect of intervention (and a penalized B-spline approximated baseline hazard). Joint models were used to obtain parameter estimates of both the longitudinal and the survival processes as well as the associations between these 2 processes.

Point estimates and 95% credible intervals (hereafter referred to as confidence intervals / CIs) of the indirect hazard ratios (HRs) were calculated using the Markov chain Monte Carlo (MCMC) samples of the Bayesian estimates for the intervention effects on the biomarker over time and of the association between said biomarker and mortality risk. In addition to point estimates of the total, direct, and indirect effects as hazard ratios, we obtained time-dependent effects on a cumulative risk difference scale using the g-computation approach.

Plasma biomarker concentrations were log10 transformed before analysis. The variable of time was treated as increasing by 1 unit per day. We adjusted for baseline covariates sex, age, body mass index, and disease severity scores in all models.

#### Meta-analysis

Since IL-6 was available in all 5 trials, a meta-analysis was conducted to synthesize the association between IL-6 concentration and mortality across studies. This was done using a random-effects model, pooling the joint model estimates of the association between IL-6 and mortality risk.

#### Software

Analyses were performed in RStudio using R version 4.4.1.^21^ The R package JMbayes2 was used for joint modeling.^22^ The meta-analysis was conducted with the R metafor package.^23^

## Results

A total of 2563 patients from 5 trials were included. Flowcharts depicting patient exclusions are available in Figure S1.

Table 1 provides an overview of the 5 trials, including their interventions and controls; sample sizes for the present analysis; mortality rates; timepoints for plasma biomarker measurements; measured pro-inflammatory biomarkers; and allocated subgroups based on inflammatory phenotypes. Baseline clinical data of patients for each trial are depicted in Table S2. All joint modeling results are included in Table S3.

**Table 1:** Overview of included studies.

Trial	Population	Intervention	Control	Phenotype subgroup	No. included	Events (%)	Days with plasma biomarkers measured	Biomarkers measured
**COUNTER-COVID**	COVID-19	Imatinib	Placebo	—	331	37 (11.2)	0, 2, 3, 5	IL-6, IL-8, TNFR1
**SAVE-MORE**	COVID-19	Anakinra	Placebo	—	587	26 (4.4)	0, 3, 6	IL-6, CRP
**ARMA**	ARDS	Low VT	High VT	—	603	183 (30.3)	0, 3	IL-6, IL-8, TNFR1
**ALVEOLI**	ARDS	High PEEP	Low PEEP	—	530	118 (22.3)	0, 3	IL-6, IL-8, TNFRI
				Hypo-inflammatory	391	62 (15.9)		
				Hyper-inflammatory	139	56 (40.3)		
**HARP-2**	ARDS	Simvastatin	Placebo	—	512	126 (24.6)	0, 3	IL-6, IL-8
				Hypo-inflammatory	335	57 (17)		
				Hyperinflammatory	177	69 (39)		

Abbreviations: ARDS, acute respiratory distress syndrome; CRP, C-reactive protein; PEEP, positive end–expiratory pressure; TNFR1, tumor necrosis factor receptor 1; VT, tidal volumes.Trials:  COUNTER-COVID: Oral imatinib to prevent pulmonary vascular leak in Covid19 – a randomized, double-blind, placebo controlled, clinical trial in patients with severe Covid-19 disease; SAVE-MORE: suPAR-guided anakinra treatment for validation of the risk and early management of severe respiratory failure by Covid-19: the SAVE-MORE double-blind, randomized phase III confirmation trial;  ARMA: Acute Respiratory Distress Network (ARDSNet) Studies 01 and 03 Lower versus higher tidal volume; ALVEOLI: Acute Respiratory Distress Network (ARDSNet) Study 04 Assessment of Low tidal Volume and elevated End-expiratory volume to Obviate Lung Injury; HARP-2: Hydroxymethylglutaryl-CoA reductase inhibition with simvastatin in Acute lung injury to Reduce Pulmonary dysfunction trial. The em dash indicates that no differentiation is made between phenotypes in the corresponding table row, i.e., no subgrouping.

### Biomarker response to intervention

**Figure 1:**
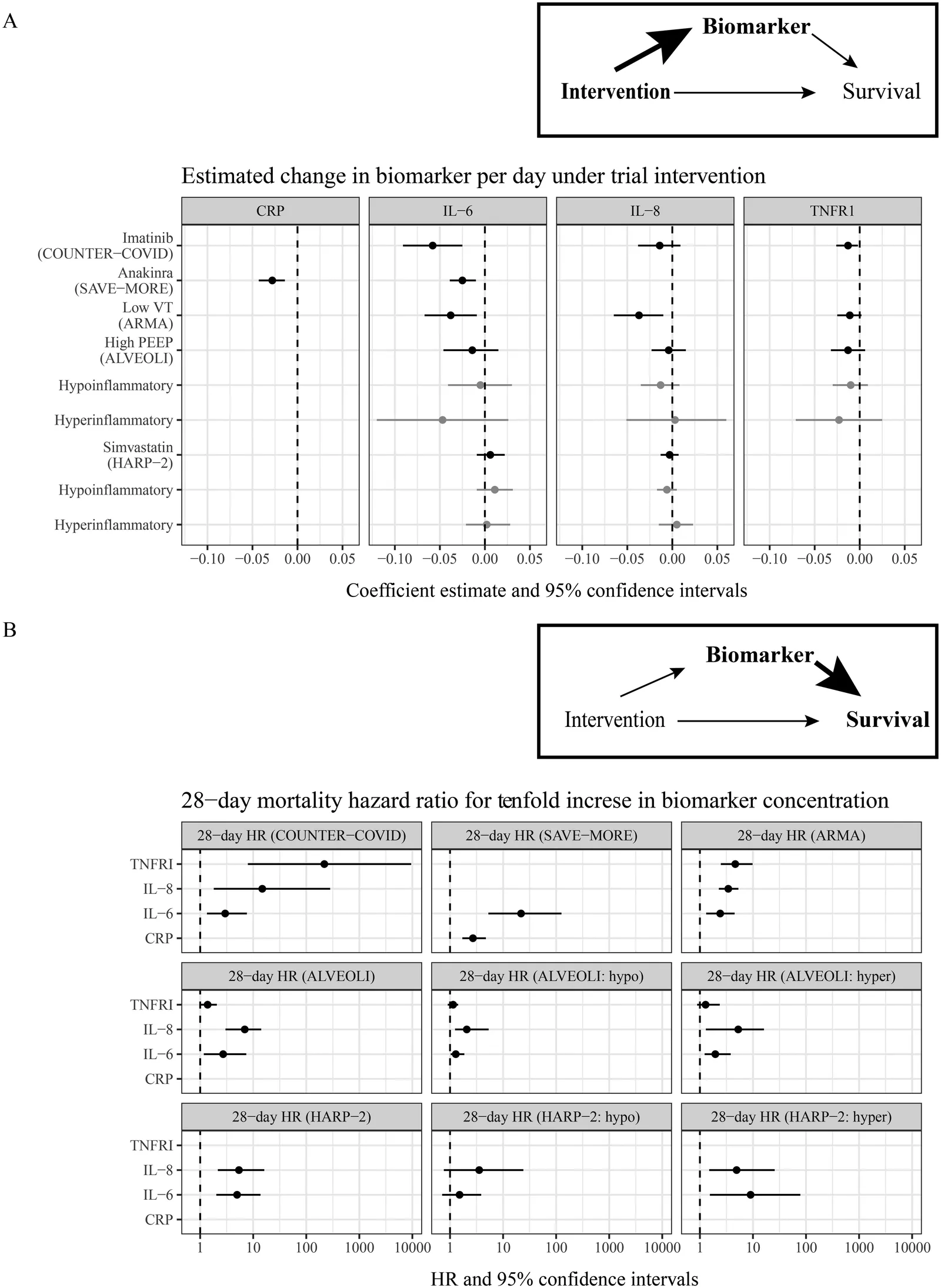
(A) Intervention effects on biomarkers over time. Illustrated by thick line in boxed figure. Estimates and 95% confidence intervals were obtained using joint modeling with a linear mixed effects model for each biomarker in each trial. The coefficients represent the estimated change in log10 plasma concentration per day under treatment. This corresponds to the interaction effect between time and treatment. (B) Associations between biomarkers and the 28-day hazards of death. Illustrated by thick line in boxed figure. Estimates were obtained using joint modeling to link biomarker values to mortality risk. The hazard ratios (HRs) represent the estimated multiplicative change in the hazard of death if the biomarkers increase 10-fold (by 1 log10 unit). Abbreviations: CRP, C-reactive protein; TNFR1, tumor necrosis factor receptor 1.

Figure 1A depicts the joint model estimates of the relationship between the trial interventions and biomarker concentrations. Imatinib reduced IL-6 by 0.06 (95% CI, -0.09 to -0.03) log10 (pg/mL) units per day, signifying a decrease of ([1−10^−0.06^] × 100) 12.91%. TNFR1 responded to imatinib with a 3% reduction. There was no significant reduction in IL-8 with imatinib (0.01, 95% CI, -0.04 to 0.01). Anakinra reduced CRP an estimated 6.24% and IL-6 with 5.60% per day. Ventilation with low tidal volumes reduced IL-6 with 8.38% and IL-8 with 8.17%. High PEEP ventilation and intervention with simvastatin did not significantly change either of the measured pro-inflammatory markers IL-6 and IL-8.

### Associations between biomarkers and mortality hazards

Figure 1B shows the joint model estimates of the associations between biomarker concentrations and the hazards of death for each respective RCT population. The hazard ratios represent the multiplicative increase in the hazard of death over 28 days when a biomarker increases 10-fold (ie, by 1 log10 unit). For all measured pro-inflammatory markers, a 10-fold increase is associated with an increased hazard of death in the 5 trial patient populations. Results for the hyper- and hypo-inflammatory subgroups of the ALVEOLI and HARP-2 trial have limited interpretability due to the strong inbuilt association of phenotype allocation to biomarkers at baseline.

### Meta-analysis: association between IL-6 concentration and 28-day mortality

**Figure 2:**
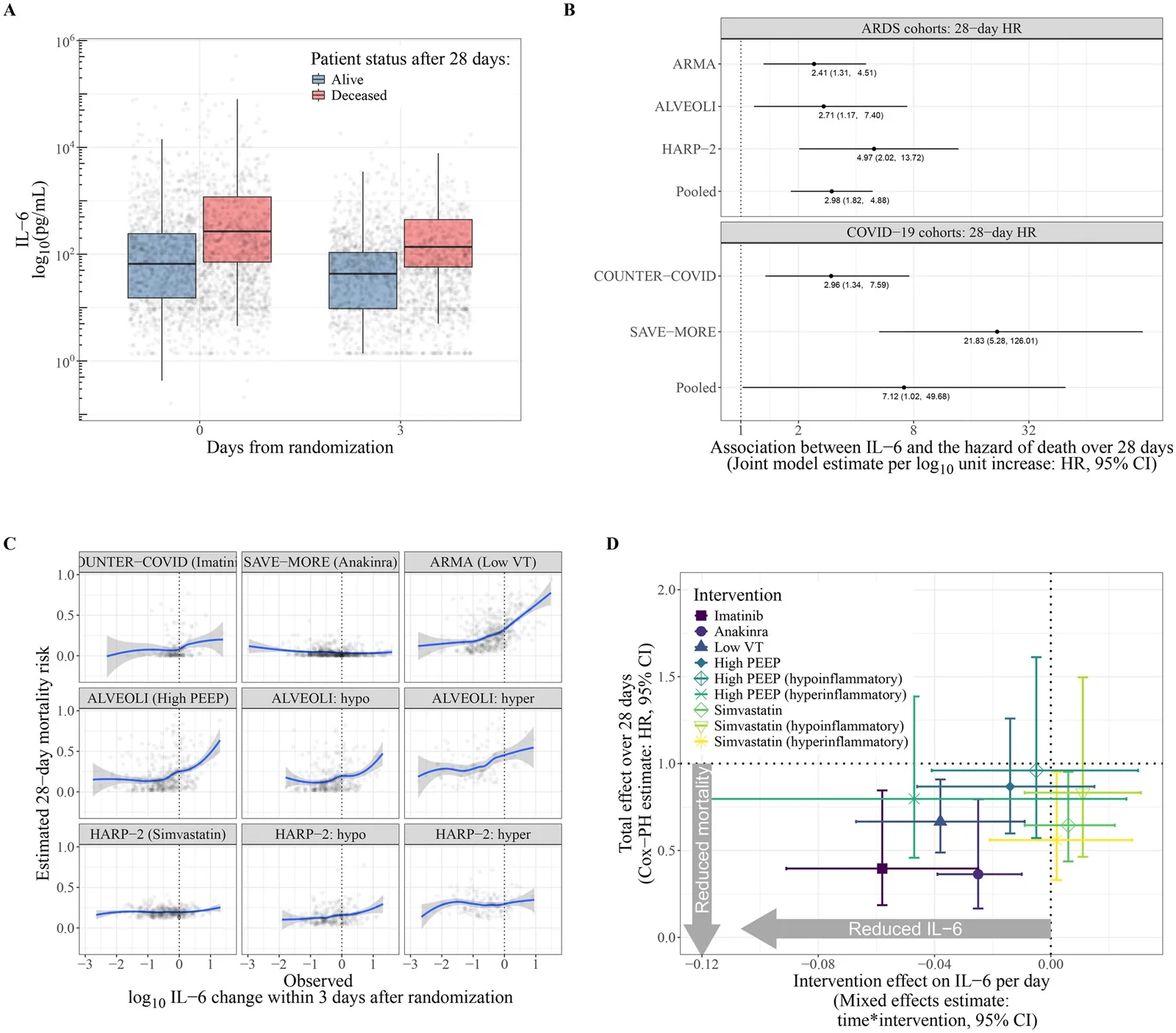
(A) Interleukin-6 concentration of all 2563 patients at baseline and 3 days after random assignment by patient status after 28 days, aggregated over all trials. (B) Meta analysis results for the association between IL-6 concentration and mortality risk. Based on the joint model estimates of the hazard ratios for mortality of a 1-unit increase in log10 IL-6 concentrations in each trial population. The hazard ratios correspond to a 10-fold increase in IL-6 concentration. The pooled estimate does not include subphenotypes for 2 reasons: (1) to keep equal weight between trials and (2) phenotype allocation is strongly associated with IL-6 concentration at day 0. (C) Patient-specific mortality risks after day 3 per observed IL-6 reductions within 3 days after random assignment. Each point represents 1 patient. Individual mortality risk was estimated using the joint models for each trial. Points to the left of the vertical dotted lines indicate patients with decreased IL-6 between days 0 and 3. Points to the right of the vertical dotted lines indicate patients with increased IL-6. (D) Intervention effects on IL-6 over time (corresponding to the mixed model estimates of the interaction between treatment and time) and total intervention effects (as hazard ratios) on 28-day mortality. Points in the lower left section represent interventions that reduce both mortality and IL-6 over time. Vertical lines around the point estimates represent 95% confidence intervals for the total intervention effect on 28-day mortality. Horizontal lines around the point estimates represent 95% confidence intervals for the intervention effects on IL-6 concentration over time. Abbreviations: ARDS, acute respiratory distress syndrome; HR, hazard ratio; PEEP, positive end–expiratory pressure.

Because only IL-6 was available in all trials, we constrained the meta-analysis to the association between IL-6 and mortality risk. We show boxplots of observed IL-6 concentrations at baseline and on day 3 after random assignment in Figure 2A. Results of the meta-analyzed associations between IL-6 and mortality are depicted in Figure 2B, separately for ARDS and COVID-19 cohorts. Pooled over the 3 ARDS cohorts, a 10-fold increase in IL-6 concentration was associated with a 2.98 (95% CI, 1.82-4.88) fold increase in the hazard of death over 28 days. Pooled over the 2 COVID-19 cohorts, this estimate was 7.12 (95% CI, 1.02-49.68). In Figure 2C, we show individual observed changes in IL-6 concentration between baseline and day 3 and the joint model estimated mortality risks over 28 days for each trial population. Patients who increase in IL-6 concentration seem to have higher estimated risk than those who decrease. Figure 2D depicts estimates of intervention effects on IL-6 concentration in the context of total effects for each trial. In trials where IL-6 is significantly reduced with treatment, there are also total intervention effects on mortality. However, not all interventions that effectively improved 28-day survival also reduced IL-6 concentration (ie, simvastatin in HARP-2).

### Mediation analysis for IL-6: hazard ratios

**Figure 3:**
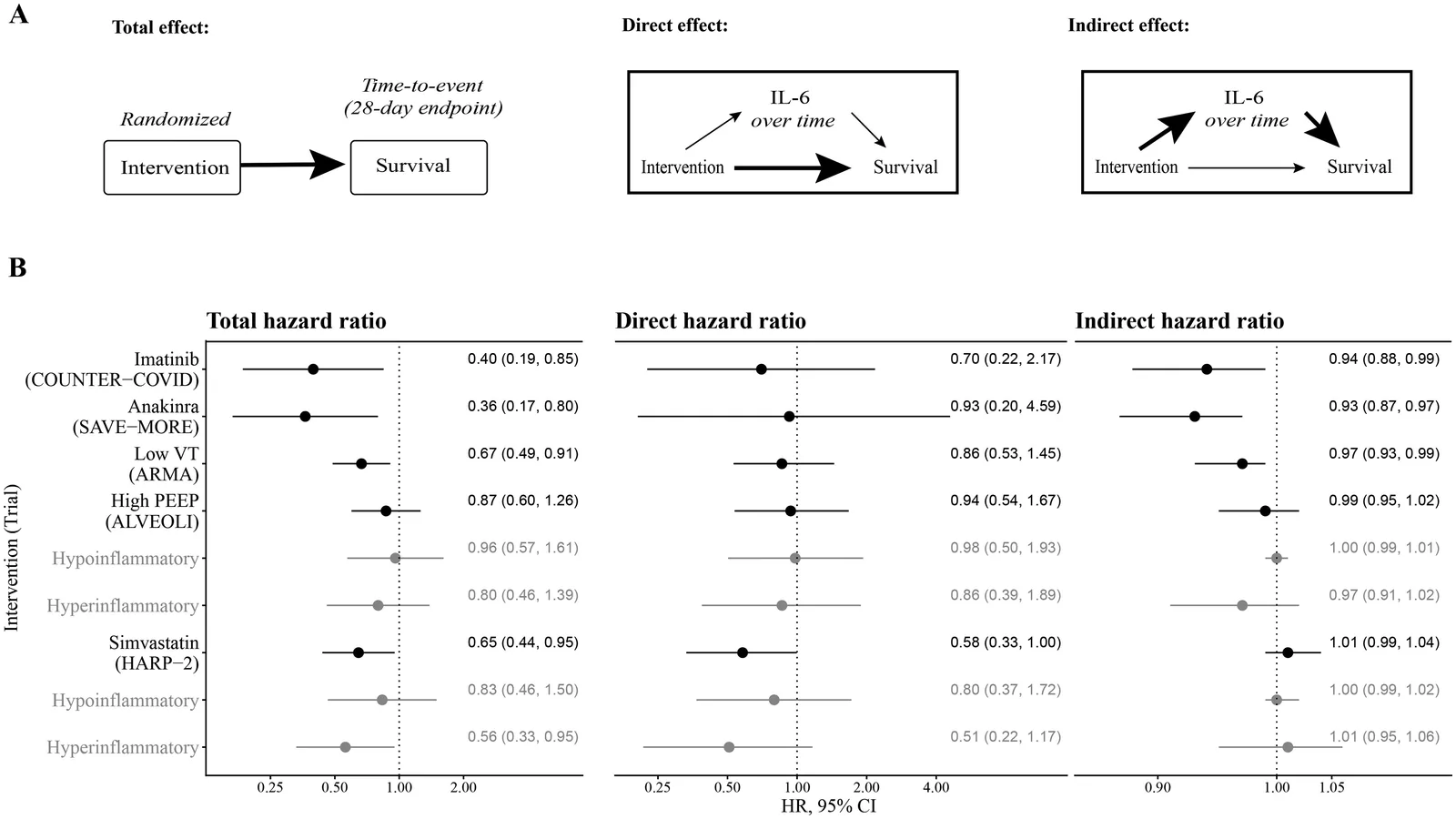
(A) Representation of the assumed mechanism where the total effect can be decomposed into a direct and an indirect effect. Direct effects represent the intervention effect on the hazard, controlling for IL-6 trajectories. Indirect effects represent the intervention effect through IL-6 modulation on the hazard. Thick arrow lines represent the effect of interest (ie, total, direct, or indirect). (B) Total, direct, and indirect hazard ratios per intervention, for ARDS studies stratified per phenotype. Interventions and controls from the randomized controlled trials: COUNTER-COVID (imatinib vs placebo), SAVE-MORE (anakinra vs placebo), ARMA (low TV vs high TV), ALVEOLI (high PEEP vs low PEEP), HARP-2 (simvastatin vs placebo). All effects are presented as hazard ratios with 95% confidence intervals for 28-day mortality. Total effects were obtained using Cox proportional hazards (PH) models. Direct and indirect effects were obtained using joint models of longitudinal and survival data, with mixed effects models of the mediator IL-6 over time and Cox PH models of the time-to-event outcome. Please note the change in *x*-axis scaling for the total hazard ratios and the direct and indirect hazard ratios. Abbreviations: ARDS, acute respiratory distress syndrome; HR, hazard ratio; PEEP, positive end–expiratory pressure.

The results of evaluating IL-6 as a mediator of treatment response are summarized in Figure 3 as hazard ratios. Here, total, direct, and indirect effects are presented as point estimates with 95% confidence intervals, adjusted for baseline covariates. The total hazard ratios are the Cox proportional hazard estimates. The direct and indirect hazard ratios were derived from joint model estimates of IL-6 response to treatment and associations between IL-6 and mortality hazards.

#### Total intervention effects on 28-day mortality

In total, imatinib reduced the hazard by 60% in comparison to the placebo (HR = 0.40; 95% CI, 0.19-0.85). Anakinra reduced the hazard by 64% in comparison to the placebo (HR = 0.36; 95% CI, 0.17-0.80). Ventilation with lower tidal volume (6 ml/kg) compared to higher tidal volume (12 ml/kg) reduced the hazard by 33% (HR = 0.67; 95% CI, 0.49-0.91). The hazard ratio for higher vs lower PEEP was neither significant in the overall cohort (HR = 0.87; 95% CI, 0.60-1.26) nor in the molecular phenotypes (hyper-inflammatory HR = 0.80; 95% CI, 0.46-1.39; hypo-inflammatory HR = 0.96; 95% CI, 0.57-1.61) groups. Use of simvastatin vs a placebo reduced the hazard in the overall cohort by 35% (HR = 0.65; 95% CI, 0.44-0.95). This reduction was not significant for the hypo-inflammatory subgroup (hypo-inflammatory HR = 0.83; 95% CI, 0.46-1.50). In the hyperinflammatory group, simvastatin reduced the hazard by 46% (hyperinflammatory HR = 0.56; 95% CI, 0.33-0.95).

#### Indirect and direct effects

The indirect hazard ratios in Figure 3 signify the change in the hazard of death under intervention that are associated with decreases in IL-6 concentration under intervention. In COVID-19 patients, the indirect hazard ratio for imatinib was 0.94 (95% CI, 0.88-0.99), indicating that a 6% reduction in mortality risk with imatinib could be explained by the IL-6 pathway. For anakinra, the indirect pathway through IL-6 explained a 7% reduction in risk. In patients with ARDS not due to COVID-19, ventilation with lower tidal volume caused a decrease in IL-6, which was associated with a 3% reduction in mortality risk. Ventilation with higher PEEP did not alter IL-6 trajectories over time in the overall cohort or in either molecular phenotype (Figure 1A), which means there was no meaningful indirect pathway (Figure 3), though higher IL-6 concentrations were associated with increased hazard (Figure 1B). Similarly, treatment with simvastatin did not change IL-6 concentrations over time in the overall cohort or in the molecular phenotype groups, indicating no indirect simvastatin-IL-6-mortality pathway. We emphasize that causal interpretation of these results should largely be avoided due to the observational nature of the biomarker measures. None of the estimated direct hazard ratios reached significance when changes in IL-6 concentration were accounted for.

### Mediation analysis for IL-6: cumulative risk differences

**Figure 4:**
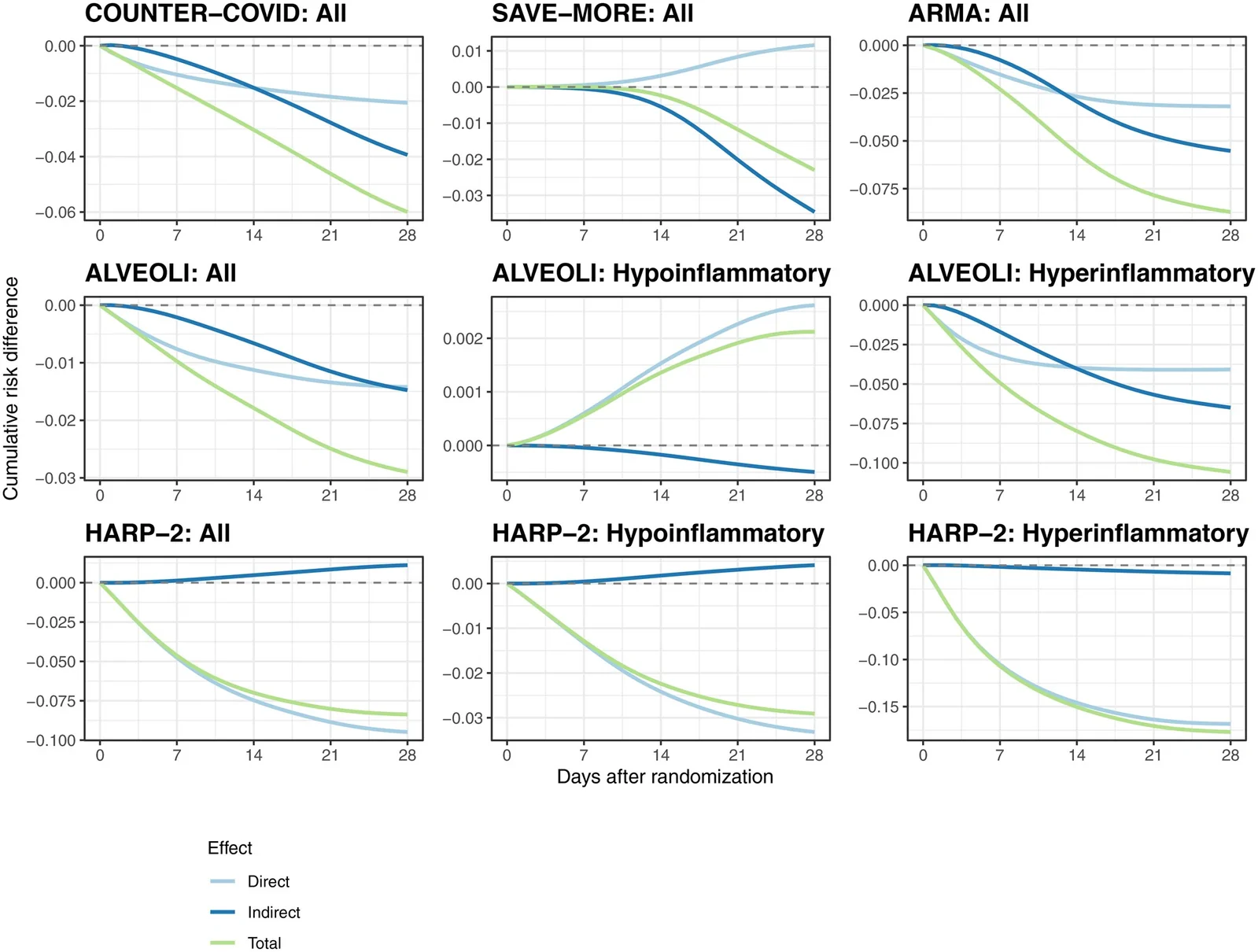
Estimated time-dependent total, direct, and indirect effects on a cumulative risk difference scale. Obtained using the g-computation approach (detailed in Supplement B). Under the framework for natural direct, indirect, and total effect, we estimated the cumulative risk differences over 28 days between intervention and control group that were attributable to the indirect pathway through IL-6 and the direct pathway not through IL-6.

Estimates of time-dependent total, direct, and indirect risk differences are presented in Figure 4. We estimated cumulative risk differences over 28 days between intervention and control group that were attributable to total pathway, the indirect pathway through IL-6, and the direct pathway not through IL-6. For a nuanced representation of these estimates, we refer the reader to supplemental Figure S5, where we provide context for absolute risk differences using Kaplan–Meier curves and show observed IL-6 trajectories for each trial. Time-dependent mediation results for IL-8, TNFR1, and CRP are included in Figure S8.

### Secondary analysis: 90-day endpoint

Similar results were observed for the total, direct, and indirect effect in the 90-day survival analysis, included in Figure S4. The total effects of the intervention on survival in SAVE-MORE and COUNTER-COVID trials could be attributed to reduction of IL-6. There was no significant total effect of low tidal volume ventilation on 90-day survival.

## Discussion

In this study, we evaluated the role of pro-inflammatory plasma biomarkers (IL-6, IL-8, CRP, and TNFR1) in treatment response, leveraging clinical and biological individual patient data from 5 RCTs for acute lung injury. Moreover, we evaluated IL-6 specifically as a potential mediator of treatment effects. The interventions imatinib, anakinra, and low tidal volume ventilation resulted in improved survival, and our results suggest this effect may partially operate through reduction of IL-6; the results were consistent with mediation. Interventions with higher PEEP and simvastatin did not alter IL-6 trajectories, suggesting no mediation in 2 of the 3 included ARDS trials. In all study patient populations there were associations between higher IL-6 and higher mortality. When controlling for IL-6 trajectories, there was no evidence for direct intervention effects on mortality risk over 28 days.

In our meta-analysis, the association between IL-6 measured during the first days after study inclusion and patient mortality was consistent between trials. Such compatible results may be surprising given that trials used various assays for IL-6 measurements, were performed decades apart, and included different patient populations with acute respiratory failure. Interleukin-6 is a pleiotropic pro-inflammatory cytokine and is involved in various inflammatory pathways.^24^ Its half-life is relatively short, when for example compared to CRP—a downstream acute phase protein that is frequently measured in clinical practice, which means that it provides accurate information of the very recent inflammatory response. This qualifies IL-6 as a reasonable prognostic biomarker in critically ill patients with ARDS and COVID-19.

While the association between IL-6 and patient outcomes was consistent across lung injury cohorts, the responsiveness of IL-6 to interventions was not. In COVID-19 patients, treatment with imatinib and anakinra reduced plasma IL-6 concentration. In ARDS patients, reductions in IL-6 concentrations occurred with low tidal volume ventilation but not with high PEEP ventilation or simvastatin. This means IL-6 is not a mediator for the effectiveness of these interventions. This may be explained by other mechanisms underlying the intervention, which are not through reduction of inflammation. However, we cannot rule out the possibility of disease-specific differences between ARDS and COVID-19 patients.

While our study included COVID-19–associated ARDS and classical ARDS patients, the proportion of COVID-19 patients may still limit generalizability to other ARDS etiologies. Although COVID-19–associated ARDS may differ from non–COVID-19 ARDS in its temporal trajectory and inflammatory profile, IL-6 is not unique to COVID-19–related inflammation. In sepsis-induced ARDS, the release of IL-6 contributes to loss of alveolar-capillary barrier integrity, neutrophil recruitment, and alveolar edema,^25^^,^^26^ and persistently elevated plasma IL-6 is among the strongest predictors of mortality across ARDS etiologies.^27^^,^^28^ Moreover, the hyperinflammatory subphenotype of ARDS is characterized by elevated IL-6 and other pro-inflammatory biomarkers, has been consistently identified across diverse ARDS trial cohorts including sepsis- and pneumonia-associated ARDS, and is associated with worse outcomes and differential treatment response.^5^^,^^29^ A comparison of cytokine profiles found that plasma IL-6 levels in severe COVID-19 did not significantly differ from those in non–COVID-19 ARDS and sepsis.^30^ The mediating role of IL-6 we identified is therefore biologically plausible beyond the current study population, though replication in heterogeneous, non–COVID-19 ARDS cohorts is warranted.

Through mediation analysis, we were able to disentangle the associations between multiple interventions and mortality. Interestingly, none of the interventions in any of the included trials exhibited a direct effect on 28-day mortality after adjusting for changes in the IL-6 concentration in plasma. Instead, we identified significant mediation of the benefit of imatinib, anakinra, and lower tidal volume through IL-6 reduction. This implies that the benefits of these interventions may, at least partly, be attributed to reduction in plasma IL-6 concentration. This does not imply that IL-6 is the one and only “magic bullet” pro-inflammatory biomarker, which is affected by intervention and associated with patient outcomes. For instance, our analysis suggested that CRP was an additional mediator for the effectiveness of anakinra in the SAVE-MORE trial, and in the ARMA trial, IL-8 partially mediated the effectiveness of low tidal volume ventilation. However, IL-6 is routinely measured, and the present results suggest that, of the measured biomarkers, IL-6 was most consistently associated with effective intervention. For an immunomodulator like anakinra, this inflammatory pathway is in line with its mode of action through the reduction of inflammasome-related inflammation. Imatinib is not generally considered to be an immunomodulator, even though broad anti-inflammatory effects have been described previously.^31^ Lower tidal volume ventilation protects against ventilator-induced lung injury partly through a reduction in biotrauma,^32^ and our results suggest that this is central to the reduction in mortality. Moreover, the protective effects observed for a high PEEP strategy in the hyperinflammatory phenotype were consistent with the reduction in IL-6 concentration in plasma, further supporting the hypothesis that reduction of ventilator-induced lung injury may be negotiated by a reduction in biotrauma.

Although our results support the potential use of IL-6 for monitoring treatment effectiveness in COVID-19 and ARDS, we emphasize that this does not imply that lowering IL-6 is a therapeutic target. In COVID-19, the IL-6 receptor antagonist tocilizumab was found to reduce mortality,^8^ despite a rise in circulating IL-6 concentrations due to compensatory pathways after loss of its receptor.^33^ The effectiveness of tocilizumab supports the notion that blockade of the IL-6 pathway is instrumental in recovery, and we present IL-6 as a surrogate for activation of this pathway, in the absence of direct interference through blocking antibodies of its receptor.

The major strength of this paper is the combination of data from multiple RCTs and sequential biological data to obtain unbiased mediation effects and pooled estimates of the prognostic value of plasma IL-6 concentration. Joint modeling is a relatively novel statistical framework particularly suitable for mediation analysis with a time-dependent mediator, and this paper may serve as an example for similar clinical challenges. A major advantage of the joint modeling approach in this study is that it allowed us to account for informative missingness of IL-6 measures due to patient death over time. However, for some patients there is a lack of IL-6 measures because they were discharged shortly after admission, which is a potential source of bias that we could not account for in our analyses. Another limitation of the present approach is that the indirect and direct effects have subject-specific interpretations that are conditional on the random effects in each trial population. Strictly, these effects do not have a causal interpretation on the population level. This is rooted in the assumed shared random effects structure of the joint model.^34^ An additional issue that prevents causal interpretation is that the present retrospective approach cannot fully account for potential confounding of the IL-6–mortality relationship. This is because IL-6 is observed, not randomized, and in the context of this mediation analysis we cannot account for postrandomization confounders that are affected by the intervention, such as driving pressure in the mechanical ventilation trials, without blocking part of the intervention effects on patient outcomes. Only a prospective trial targeting modulation of IL-6 would be able to prove causality of the relationship between IL-6 and patient outcomes.

One potential limitation of our study is that not all patients in the included COVID-19 trials may meet the classical Berlin definition of ARDS. However, under the recent Global Definition of ARDS,^35^ which broadened diagnostic criteria to include an SpO_2_/FiO_2_ ratio ≤ 315 when SpO_2_ ≤ 97%, approximately 80% of COUNTER-COVID patients at inclusion fell below this threshold, consistent with at least mild ARDS. We therefore use the term *lung injury* where appropriate to reflect the heterogeneity of respiratory failure in our study population while noting that the severity of hypoxemia in our cohorts aligns with clinically significant acute lung injury across the included trials.

Another challenge was the lack of a standardized IL-6 assay between cohorts, which may have increased measurement error. Biomarker data were measured at particular timepoints as a result of sampling in the trials, and it remains unclear how the true trajectories of individual patients progressed during the time between the last biomarker observation and the survival endpoint. We had to assume a linear change in biomarker trajectories over time, which may very well be a simplification of reality. Furthermore, some of the effects have large confidence intervals. Most noticeable in the presented analysis results of the SAVE-MORE data, this is due to only a small number of patients experiencing the event, which limits power. The large estimate of the association between IL-6 and mortality risk in the SAVE-MORE patient population should therefore be treated with caution. The issue of small samples also pertains to the subgroup analyses of classified hypo- and hyperinflammatory patients. Only a minority of patients are classified as exhibiting a hyperinflammatory molecular phenotype, while this is also the subgroup of patients that may experience most benefit of interventions. The sample sizes in this re-analysis of trial data were further shrunken because we could only include patients with available IL-6 measures. In the re-analysis of the ALVEOLI trial, this extinguished any evidence for a benefit of mechanical ventilation with high PEEP, even in the classified hyperinflammatory subgroup.

The implications of this study are 2-fold. First and foremost, IL-6 should be further explored as a central mediator of lung injury. This single biomarker may need to be integrated with other data. We showed that IL-6 is a mediator of therapy effectiveness, which suggests it may be useful to guide therapy. For example, future research might aim to use nonresponse of patients’ IL-6 concentration to titrate therapies. Recently, we showed that resolution of the probability of a hyperinflammatory phenotype is of strong prognostic value irrespective of organ dysfunction, illustrating such an approach.^36^ Secondly, the underlying inflammatory pathways resulting in decline in IL-6 concentrations over time need to be understood better, as this could not be distilled from the available biological data in the included RCTs. Another implication of this study is that a joint modeling approach can be used to obtain mediation effects while accounting for informative missingness, which will be pivotal to further our understanding of recovery pathways in critical illness in general.

The present study suggests that resolution of inflammation, as reflected by plasma IL-6 concentrations, may explain effectiveness of interventions in patients with COVID-19 or ARDS. Interleukin-6 might offer value in understanding treatment mechanisms and guiding trial designs. With growing availability of bedside biomarker measurements, and potential prospective studies evaluating the relationship between IL-6 and patient outcomes, IL-6 could serve as a surrogate marker for treatment effectiveness in clinical trials.

## Supplementary Material

aamag213_Supplementary_Data

## Data Availability

All R code used for analysis is available under github.com/linakramerr/ards. This repository also includes a simulated example dataset which is used to illustrate the employed mediation analysis approach.
