# Supplementary material for: Interleukin-6 is a mediator of therapeutic efficacy in acute lung injury
Source: Am J Respir Crit Care Med. 2026 Apr 28;212(8):1750–60. doi: 10.1093/ajrccm/aamag213 (PMC13424682; doi:10.1093/ajrccm/aamag213)
Supplement: aamag213_Supplementary_Data [file aamag213_supplementary_data.zip › coi_disclosure 032326 Calfee.docx]

**ICMJE DISCLOSURE FORM**

**Date:_** 3/23/2026

**Your Name:** Carolyn S Calfee MD

**Manuscript Title:_** Interleukin-6 is a mediator of therapeutic efficacy in acute lung injury

**Manuscript number (if known):**

**In the interest of transparency, we ask you to disclose all relationships/activities/interests listed below that are**

**related to the content of your manuscript. “Related” means any relation with for-profit or not-for-profit third**

**parties whose interests may be affected by the content of the manuscript. Disclosure represents a commitment**

**to transparency and does not necessarily indicate a bias. If you are in doubt about whether to list a relationship/activity/interest, it is preferable that you do so.**

**The following questions apply to the** **author’s relationships/activities/interests as they relate to the current**

**manuscript only.**

**The author’s relationships/activities/interests should be defined broadly. For example, if your manuscript pertains**

**to the epidemiology of hypertension, you should declare all relationships with manufacturers of antihypertensive medication, even if that medication is not mentioned in the manuscript.**

**In item #1 below, report all support for the work reported in this manuscript without time limit. For all other items,**

**the time frame for disclosure is the past 36 months.**

|  |  | **Name all entities with whom you have this relationship or indicate none (add rows as needed)** | | **Specifications/Comments**  **(e.g., if payments were made to you or to your institution)** |
| --- | --- | --- | --- | --- |
| **Time frame: Since the initial planning of the work** | | | | |
| 1 | All support for the present manuscript (e.g., funding, provision of study materials, medical writing, article processing charges, etc.)  **No time limit for this item.** |  |  |  |
|  |  | NIH | | Grant to my institution |
|  |  |  | |  |
|  |  |  | |  |
|  |  |  | |  |
|  |  |  | |  |
|  |  |  | |  |
|  |  |  | |  |
| **Time frame: past 36 months** | | | | |
| 2 | Grants or contracts from any entity (if not indicated in item #1 above). | Roche Genentech | Grant to my institution | |
|  |  |  |  |  |
|  |  | NIH, DOD | Grants to my institution | |
|  |  | Quantum Leap Healthcare Collaborative | Grants to my institution | |
| 3 | Royalties or licenses | ____None |  | |
|  |  |  |  | |
|  |  |  |  | |
| 4 | Consulting fees | Vasomune | Consulting fee to me | |
|  |  | Gen1e Life Sciences | Consulting fee to me | |
|  |  | NGM Bio | Consulting fee to me | |
|  |  | Cellenkos | Consulting fee to me | |
|  |  | Calcimedica | Consulting fee to me | |
|  |  | Arrowhead | Consulting fee to me | |
|  |  | EnliTISA | Consulting fee to me | |
|  |  | Novartis | Consulting fee to me | |
|  |  | Merck | Consulting fee to me | |
|  |  | Healios | Consulting fee to me | |
|  |  | Matisse | Consulting fee to me | |
|  |  | Boehringer | Consulting fee to me | |
| 5 | Payment or honoraria for lectures, presentations, speakers bureaus, manuscript writing or educational events | Fisher-Paykel | Supported a symposium at which I spoke on ESICM guidelines | |
|  |  |  |  | |
|  |  |  |  | |
| 6 | Payment for expert testimony | ____None |  | |
|  |  |  |  | |
|  |  |  |  | |
| 7 | Support for attending meetings and/or travel | ____None |  | |
|  |  |  |  | |
|  |  |  |  | |
| 8 | Patents planned, issued or pending | Patent on metagenomic sequencing for sepsis diagnosis (co-recipient) | Issued to Regents of University of California and Chan Zuckerberg BioHub | |
|  |  |  |  | |
|  |  |  |  | |
| 9 | Participation on a Data  Safety Monitoring Board or Advisory Board | ____None |  | |
|  |  |  |  | |
|  |  |  |  | |
| 10 | Leadership or fiduciary role in other board, society, committee or advocacy group, paid or unpaid | International Sepsis Forum | Council member (unpaid) | |
|  |  |  |  | |
|  |  |  |  | |
| 11 | Stock or stock options | ____None |  | |
|  |  |  |  | |
|  |  |  |  | |
| 12 | Receipt of equipment, materials, drugs, medical writing, gifts or other services | ____None |  | |
|  |  |  |  | |
|  |  |  |  | |
| 13 | Other financial or non-financial interests | ____None |  | |
|  |  |  |  | |
|  |  |  |  | |

**Please place an “X” next to the following statement to indicate your agreement:**

**__x_ I certify that I have answered every question and have not altered the wording of any of the questions on this**

**form.**
